# The Effects of Multifaceted Ergonomic Interventions on Musculoskeletal Complaints in Intensive Care Units

**DOI:** 10.3390/ijerph17103719

**Published:** 2020-05-25

**Authors:** Ayse Coskun Beyan, Banu Dilek, Yucel Demiral

**Affiliations:** 1 Occupational Medicine Department, Dokuz Eylul University, Izmir 35220, Turkey; yucel.demiral@deu.edu.tr; 2 Physical Therapy and Rehabilitation Department, Dokuz Eylul University, Izmir 35220, Turkey; banu.dilek@deu.edu.tr

**Keywords:** ergonomics, intensive care nurses, patient lifting system, Cornell musculoskeletal discomfort questionnaire, work-related diseases

## Abstract

Working at intensive care units (ICUs) is considered a risk factor for developing musculoskeletal complaints (MSC). This study was conducted between January 2017 and June 2019 in two ICUs of a university hospital. It was designed as a pre- and post-assessment of the intervention group (IG) (N = 27) compared with a control group (CG) (N = 23) to determine the effects of a multifaceted ergonomics intervention program in reducing MSC. The IG (N: 35) received a multifaceted ergonomic intervention program, which was implemented by an ERGO team over an 18 month period. Four ergonomic interventions were planned as follows: individual level interventions such as training; stretching exercises and motivation meetings; administrative intervention such as a daily 10 min stretching exercises break; engineering interventions such as lifting and usage of auxiliary devices. The CG (N:29) did not receive any intervention. Cornell Musculoskeletal Discomfort Questionnaire (CMDQ) was used to assess MSC in both groups. At the start of the intervention, both groups were similar concerning the number of visits to doctors due to MSC, the number of sick leave days, and total CMDQ scores (*p* > 0.05 for all). Two factor repeated ANOVA measures were performed for between-groups and within-group analyses. The mean of the initial CMSDQ total scores in both groups increased significantly in the 18th month (*p* < 0.001). However, the interaction effect of group and time (between and within factors) was not significant (*p* = 0.992). Work-related MSC is a common occupational health problem among nurses. This study showed that individual-level interventions are not likely to succeed in eliminating manual patient lifting by nurses. Our results suggested that interventions without administrative measures might have limited success

## 1. Introduction

Musculoskeletal diseases (MSD) are one of the most common work-related health problems. Repetitive trauma, cumulative strain, or acute injury may cause MSD arising when the self-healing and repair capacities of affected structures have been exceeded [1]. Intensive care units (ICUs) are noted as having one of the highest occupational risks in terms of ergonomic factors. Musculoskeletal complaints (MSC) can be caused by physical, psychosocial and organizational factors, such as standing for long hours, heavy lifting, working with computers, bending, transferring patients out of bed, working in awkward position, high work demands, low decision latitude, unsatisfying job content, and low social support [2]. These risk factors have been extensively studied for the development of acute and cumulative MSD [3,4,5]. The prevalence of MSC/MSD in nurses was found to be varied in the literature [6,7,8,9,10]. Tinubu et al. reported 44% low back pain and 28% neck pain prevalence in Southwest Nigerian nurses [9].

Stolt et al. reviewed 935 studies and reported that knee (prevalence from 7.5% to 77%) and ankle/foot regions (prevalence from 3.2% to 100%) were most often affected [4]. Sezgin et al. reported that the highest prevalence for MSC of nurses was in the legs, lower back, and back. The prevalence of MSD for the whole body was 95.9% [11]. Higher prevalence of back pain and occupational back injuries were reported for nurses compared to other occupational groups [12].

MSD can have important consequences on quality of life that might result in absenteeism and have a socio-economic impact on organizations. According to national data, total injury costs in the United States for nurses and nursing aides have been estimated to be 1.6 billion US Dollars for lower back in 2013 [13]. In Finland, nurses have more MSD-related sick leave days than other working populations [14]. Therefore, exploring the benefits of ergonomic interventions to protect and prevent MSC in ICU workers is of great concern in occupational health and safety services in health care settings.

Ergonomic interventions such as training, using auxiliary lifting devices, and exercise programs have been adopted in different countries, and results of controlled studies have been reported. The results are rather inconclusive and both successful and unsuccessful aspects of these programs are discussed by the authors [15,16]. Szeto et al. presented a pilot study to investigate tailored ergonomic intervention programs (training, administrative measures, equipment redesign, etc.) for community nurses with MSC. They found statistically significant improvement in the intervention group when compared to the control group [17]. Hignett systematically reviewed 2796 papers, selected 63 of them and highlighted that multidimensional interventions based on a risk assessment program would be beneficial compared to interventions focused on selected problems such as education or training [18]. Hartvigsen et al. evaluated the effectiveness of tailored ergonomic intervention programs among home care nurses and nurses’ aides, and no significant differences were found between the intervention and the control group in terms of the number of days with MSC [19]. Hegewald et al. reviewed ergonomic intervention studies which indicated that the riskiest movement for occupational musculoskeletal injuries was patient transfer. They stated that technical aids seem to have a positive effect on the prevention of musculoskeletal complaints, but the level of evidence is not very high [20].

This study was triggered by the increasing number of nurses presenting with MSC in the ICUs of our hospital. Therefore, it was planned to develop a multifaceted ergonomic risk prevention program, including the participation of the workers. It was purposed to determine the prevalence of MSC and workers’ perceptions of the ergonomic risk factors that could contribute to work-related injury, and to evaluate the effect of tailored ergonomic interventions on self-reported musculoskeletal wellbeing parameters in ICU nurses, based on ergonomic risk assessment.

## 2. Materials and Methods

### 2.1. Settings and Study Design

**Scheme 1:**
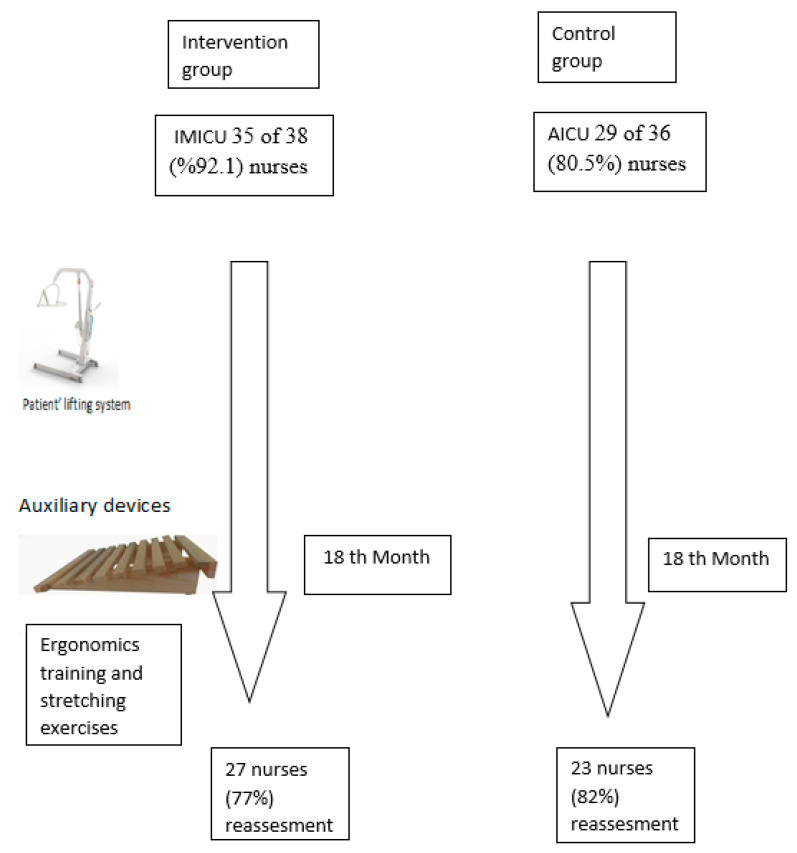
Flowchart of study.

This study was conducted between January 2017 and June 2019 as a pre- and post-assessment of the intervention group (IG) compared with a control group (CG) to determine the effects of a multifaceted ergonomics intervention program in reducing MSC. It was carried out with nurses working in ICU in the internal medicine department (IMICU-intervention group) and anesthesia department (AICU-control group) at the university hospital. There were no exclusion criteria; all registered nurses in both ICUs were intended to participate in this study. In the first evaluation, 35 of 38 (92.1%) nurses from the IG and 29 of 36 (80.5%) nurses from CG participated. The second evaluation (18th month) comprised 27 of 35 (77.1%) nurses of the IG and 23 of 29 (79.3 %) of the CG. Totally, in both units, eight nurses dropped out due to maternity leave; three nurses dropped out due to change of department. Data analysis was restricted to those nurses with assessments before and after the 18th month intervention period. The IG received a multifaceted ergonomic intervention program over 18 months (Scheme 1). The control group did not receive any intervention. The initial and follow-up assessments in both groups included musculoskeletal wellbeing parameters (Cornell Musculoskeletal Discomfort Questionnaire (CMDQ) total score, the number of sick leave days, use of medicine due to any MSD, visiting a doctor with any MSC) and perceived ergonomic risk factors.

### 2.2. Data Collection

#### 2.2.1. Questionnaire

Before the intervention, a baseline assessment of all participants in both ICUs was conducted using a questionnaire. Socio-demographic data (gender, age, information about working conditions such as total working years in ICU, participation in previous ergonomic training programs, frequency of regular exercise), as well as ergonomic risk perceptions for heavy lifting, pushing-pulling, standing for long hours, bending, and musculoskeletal wellbeing parameters were obtained.

Detailed information was obtained about the variables provided below:

- Regular exercise: Defined as doing physical activity at least three days a week or more (measured by Yes or No responses).
- Chronic disease: Defined diseases such as Diabetes Mellitus (DM) Hipertension (HT), Rheumatological diseases, etc. diagnosed by any doctor. (Please code as Yes or No and write details).
- Perceived ergonomic risk: Four items were developed based on the literature review to assess nurses ‘perceptions. Do you think there is a risk of heavy lifting/pushing-pulling/standing for long hours/bending down in your workplace? Please code as no risks/moderate risks/high risks.

Self-reported musculoskeletal wellbeing parameters:

- Visiting a doctor with any MSC: “Have you applied a doctor due to musculoskeletal complaints for the last six months?” (Measured by Yes or No responses).
- Use of medicine due to any MSD: “Have you used any medicine due to musculoskeletal complaints for the last six months? (Measured by Yes or No responses)
- Sick leave absence days: “Have you been absent in the last six months due to MSC?
- MSC: In the second part of the questionnaire, self-reported MSCs were evaluated by CMDQ. Questions of the scale were as follows: (1) How often did you encounter discomfort, pain, and/or aches when you were last at work? (Frequency score coded as 0/1.5/3.5/5/10), (2) How uncomfortable were you when you encountered such discomfort, and/or pain/aches? (discomfort/severity score coded 1/2/3). If you experienced this discomfort, pain, and/or aches, did you also experience any form of interference in your work? (Interference score coded 1/2/3). Lower back, shoulders, upper arms, upper back, neck, forearms, knees, hips, wrists, thighs, left lower leg, and right lower leg parts were evaluated separately. According to the recommendations of the developers of the scale, the total Cornell score was calculated by multiplying frequency score, discomfort score, and interference score for each body part. The total score was obtained by calculating each body part score. Higher Cornell score indicates a bad condition. There is no cut-off point of the Cornell scoring system. Cornell score differences between groups or longitudinal evaluation of the given group could also be evaluated [21]. Translation of the Cornell questionnaire, validity and reliability assessment, and cross-cultural adaptation into the Turkish language, was performed by Erdinc et al. Cornell neck, Cornell upper limb, Cornell back, and Cornell lower limb scores were calculated according to the REBA risk assessment [22].

#### 2.2.2. ERGO Team

The ERGO team was established to plan ergonomic risk assessment and implement an ergonomics program. Members of the ERGO team were occupational physicians, physical therapy and rehabilitation specialists, occupational medicine specialists, occupational health nurses, and voluntary nurses from IG.

#### 2.2.3. ERGO Program

**Figure 1:**
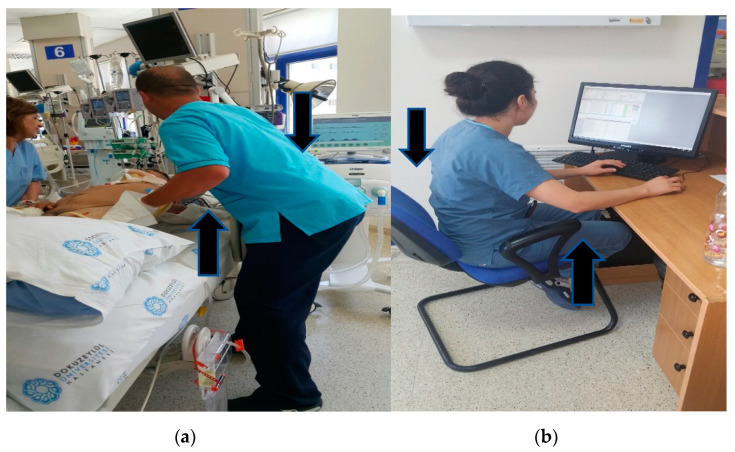
(**a**) Patient positioning and (**b**) working with the computer.

Walkthrough survey and meetings:

The ERGO team had been prepared for the assessment by interviewing the nurse and observing the nurse’s shifts. The nurse was being evaluated to obtain an understanding of each job task and its ergonomic demands. Each ICU was visited at least ten times by the ERGO team at different times. Group meetings and person-to-person meetings were held with the workers. As a result of these meetings, the two most ergonomically risky tasks were identified, namely turning the patient (Figure 1a) and working with the computer (Figure 1b).

#### 2.2.4. Ergonomics Risk Assessment

The Rapid Entire Body Assessment (REBA) ergonomics risk assessment tool was used. In the REBA method, the body is divided into two groups: the first group is neck, torso, and legs; the second group is arm, forearm, and wrist. The method consists in assigning a score to each part of the body; this gives two different scores that represent the level of postural load of the musculoskeletal system determined from the combination of the whole-body postures. The final score is between 1 and 15 and defines five risk levels. Score 1 indicated a negligible risk, no action required; score 2–3 indicated that low-risk change might be needed; score 4–7 indicated a medium risk requiring further investigations and change; score 8–10 indicated a high risk; and score >11 indicated a very high risk and the need to implement change [23,24].

In the REBA method, observation could be completed in three ways: direct observation, video recording or taking photographs to obtain data [25]. In this study the REBA risk assessment performed by two different ERGO team members with on-site direct observation. The REBA risk score calculation was gained as follows: the scores given by the two observers independently were then evaluated together, and the final score was determined. We did not perform motion capture or video analysis to find the joint angles. 

### 2.3. Interventions

Four ergonomic interventions were planned as follows:

Onsite group training: the ERGO team created a standard group training program. Training topics were defined as: ergonomic risks and prevention in ICUs, using auxiliary devices, stretching exercises, occupational MSD, and musculoskeletal injuries. The participation rate was over 80%. All training was completed within the first month of the study and was not repeated.

Auxiliary devices: According to the information obtained from REBA risk assessment, a slide sheet, and a scroll board (for each patient’s bed), underfoot support (5 pieces), and a patient lifting system (1 piece) were provided. All the workers were trained to use the devices. 

A regular stretching exercises program was implemented as follows: stretching exercises were planned for 10 min before starting each shift. Thus, each nurse planned to join at least one five-stretching exercises program per week. Each month, one exercise coach was chosen from the group and motivated to apply for the stretching exercise program through social media and phone calls. There are two means of defining exercises. First, whole body stretching exercises were tested before the research, which lasted a maximum of 10 min. The American College of Sport Medicine’s recommendation suggests that stretching should be included in an exercise program at least two to three times per week [26]. Despite the lack of a specific number of exercises, it is also recommended that two to four sets of stretching should be performed, where a given position should be held for ten 10 to 13 s until the point of slight discomfort, accumulating 60 s for each exercise, Stretching sessions should have an average duration of six to eight min in the case of two to three exercises employed for specific muscle groups [27]. Second, the nurses included in our study continued their daily work. These exercises are recommended to be performed at the workstation, and only require a few seconds to perform [28]. 

General evaluation meetings were planned every three months. There were three meetings held during the study. All the nurses in IG were invited to the meetings, and approximately 30 nurses attended each meeting. At the meetings, participants were encouraged to give feedback and overall evaluation of the interventions. Three sessions were completed during the first six months.

### 2.4. Statistics

Compliance with normal distribution was assessed with the Shapiro-Wilk test. Homogeneity of variances was controlled by Levene’s test. The degree of statistical significance has been set as *p* < 0.05. Descriptive data were given as the mean with standard deviation, median, and min-max values. The dependent variables were Cornell MSDQ and musculoskeletal wellbeing parameters described above. Categorical variables were evaluated by the chi-square test and Fisher’s exact tests. Mann–Whitney U-test was adopted for between-group comparisons at both the pre- and post-intervention stages. McNemar chi-square and Wilcoxon Signed Rank Test were used to analyze for within-group comparisons. Two-factor repeated measures ANOVA was used to compare the initial and 18th-month assessments of the Cornell MSDQ of the IG and the CG. The primary purpose of a two-factor repeated measures ANOVA is to understand if there is an interaction between these two factors on the dependent variable. We investigated the interaction effect of group and time. Due to the non-normal distribution of the Cornell MSDQ score, log 10 transformation was performed. Mean values and 95% confidence interval were presented in Two-factor repeated measures ANOVA. SPSS 21.0 package program (IBM Corp. Released 2012. IBM SPSS Statistics for Windows, Version 21.0. Armonk, NY, USA: IBM Corp.) was used for all statistical analyses.

### 2.5. Ethical Approval

Ethical approval was obtained from the Ethics Committee of the University (DEU ethical committee, number: 2016/01-05) before initiating the study, and written informed consent was obtained from each nurse.

## 3. Results

Table 1 summarizes the main characteristics of participants. IG nurses were younger with a shorter duration of employment. The nurses’ mean age was 31.06 ± 5.5 (min: 23, max: 45) years in IG and 33.6 ± 5.7 (min: 26, max: 49) years in CG, respectively. Mean working time in ICU was 6.23 ± 5.7 (min: <1 max: 24) years in IG and 8.9 ± 5.9 (min <1–max: 28) years in CG. Most of the workers were females in both units. 54% and 31% of nurses had participated in previous ergonomic training in their working life. Smoking status was distributed similarly in both units. The frequency of regular exercise is very low in both groups. While, in the IG, six (17.1%) nurses were diagnosed with chronic disease, in CG four (13.8%) nurses had a chronic disease. The most common diseases were HT and DM in both groups. At baseline, the intervention and CG were similar in terms of age, gender, smoking status, chronic diseases, ergonomics training, working time in ICUs, and regular rest break use and regular exercise (*p* > 0.05 for all). 

### 3.1. Initial Assessment of Nurses’ MSC and Ergonomic Risk Factor of ICUs

Table 2 shows musculoskeletal wellbeing and sick leave absence days of the studied nurses’ last six months for the IG and CG. The sum Cornell score mean was 141.9 ± 147.9 (min–max: 0–638) in IG versus 176.1 ± 203.6 (min–max: 0–808) in CG, respectively. There was also no significant difference between the results of Cornell neck, Cornell upper limb, Cornell back, and Cornell lower limb (*p* > 0.05 for all).

Of the 35 nurses, 15 (42.9%) had visited a doctor due to MSC, and 22 (62.9%) had use medicine (myorelaxant, analgesic) for these symptoms in IG. Similarly, among CG, 14 (48.3%) had visited a doctor, and 19 (65.5%) had used medicine due to MSC in CG.

With regard to musculoskeletal discomforts characteristics among the IG nurses, the highest complaint percentage referred to lumbar back pain (45%), knees (37%), right shoulder (36%), neck (25%), and upper right arm (24%). The complaints were similarly distributed in CG: the highest complaint percentage referred to lumbar back pain (41%), right shoulder (36%), and neck (27%). There was no statistically significant difference between the IG and CG nurses in terms of MSC in the last six months.

Table 3 presents ergonomic risk analysis results from both nurses and the ERGO team. According to the ERGO team’s observations, the mean REBA score was calculated for working with computers and turning the patients. The mean REBA scores for working with computers and turning the patients were 4.81 ± 4 in IG versus 6.11 ± 6 in CG and 8.7 + 2.0 in IG versus 9.7 + 1.6, respectively. There was no significant difference between IG nurses and CG nurses. Nurses in both ICUs were perceived to be at high ergonomic risk. The nurses were asked to determine their ergonomic risk for four common tasks/or movements in ICUs in their daily work. 80%of the 35 nurses thought they had high ergonomic risks in terms of heavy lifting, long-standing, bending down, and pulling-pushing in IG. Similarly, more than 70% of the 29 nurses thought they had high ergonomic risks in CG. Only one nurse in IG and only two nurses in CG stated that they did not have any ergonomic risks. There was no difference in self-risk perceptions before and after the intervention in both IG and CG (not given in Table 3) (*p* > 0.05 for all).

Table 4 represents the findings related to the effects of interventions on musculoskeletal discomfort and sick leave absence days for each ICUs. Totally, in both units, eight nurses dropped out due to maternity leave; three nurses dropped out due to change of department. According to within-group analysis of nurses’ scores, there was no significant difference found between musculoskeletal wellbeing parameters. While the number of nurses who visited a doctor with any MSC was 16 (59.3%) at the initial assessment of the study, in the IG it decreased to 13 (56.5%) in the 18th month. Similarly, it decreased in CG. There was no statistically significant difference in the within-group analysis (*p*: 0.75 and 0.06).

Table 5 states the between-group analysis in the 18th month. At the last evaluation, the intervention and CG were similar in terms of visiting any doctor due to MSC, the number of sick leave absence days, and self-ergonomic-risk assessment in terms of heavy lifting, long-standing, bending down and pulling-pushing. There was an increase in ergonomic risk perception in both groups. In the initial assessment, 80% of the nurses evaluated a high risk for long-standing, while in the 18th-month evaluation this rate increased to 96.3% in IG (not statistically significant) This increased in the CG from 79.3% to 87% (not statistically significant). Similar to other ergonomic risks, there is an increase in the high-risk assessment rate, but there is no statistical significance in the within group analysis and between-group analysis of both groups (not given in the tables). 

According to two-factor repeated measures ANOVA, the mean of the initial Cornell MSDQ total scores in both groups increased significantly in the 18th month (*p* < 0.001). However, the interaction effect of group and time (between and within factors) is not important (*p* = 0.992). There is no statistically significant difference between the groups (*p* = 0.641). Both the IG and CG reported musculoskeletal discomforts in similar body regions at post intervention (Table 6). For the IG and CG, the initial four regions with the highest complaint percentage referred to lumbar back pain, knees and right shoulder, neck, and upper right arm, similarly. The CG reported the same most common areas, except that the fourth was the elbow region and not the upper right arm. It was remarkable that Cornell scores increased in both ICUs. However, the total Cornell score of 10 nurses working in IG decreased compared to the previous evaluation, while in the CG six nurses improved (*p*:0.034) (Not shown in the table).

### 3.2. Nurses Participation in Study and Compliance with Continuity of Interventions

The highest attendance and compliance were in ergonomics training. Training was completed in four sessions within the first one month (participation rate was over 80%). The most important adaptation problem in the use of auxiliary devices was experienced in the patient lifting system. Two main reasons were indicated by the nurses that explain incompatibility:

- Having only one device that needs to be carried and installed over and over again for each patient.
- Especially for the first time, taking too much time to install.

Nurses were encouraged to perform stretching exercises together at certain times. We tried to keep motivation high through social media and handbooks. However, initially high compliance decreased over time, especially in the last six months. The insufficient number of workers in the hospital and time pressure led to a lack of motivation towards stretching exercises, especially during the last stages of the research.

## 4. Discussion

Multifaceted ergonomics intervention programs are commonly used in ICUs to decrease occupational MSD and MSC. The results of this study show that interventions without administrative measures may have limited success, even if nurses have a high awareness of ergonomic risks. 

Ergonomics programs on prevention and reducing strategies for MSD are recommended for nurses to reduce the rate of work-related ergonomics risks, and results of controlled studies have varied in the literature [29,30]. Hartvigsen et al. evaluated the effectiveness of tailored ergonomic intervention programs among home care nurses and nurses’ aides with a study design similar to our study. In the intervention group, nurses and nurses’ aides were divided into small groups. One nurse or nurses’ aide was trained as an instructor for each group. This program continued for a minimum of one hour per week for two years to educate, supervise, and enforce messages about lifting techniques and body mechanics to all members of the group. At follow up, no significant differences were found between the intervention and the control group in terms of the number of days with MSC. Similar to our results, in the intervention group complaints improved slightly, but there was no statistically significant difference. This highlighted that the program was implemented efficiently. After these reviews, the authors concluded that the results of this study are most likely due to a basic lack of effect of these ergonomic interventions [19]. Our results also supported that the interventions, including training, assistive devices, and stretching exercises, had limited effects on MSC.

Hignett discussed the results of a systematic review looking at occupational hygiene implementation strategies to reduce the ergonomic risk factors associated with patient care activities. There is strong evidence that interventions predominantly based on technique training have no impact on working practices. Multifaceted interventions, based on a risk assessment program, engineering, devices, work environment redesign, education and training, and changes in work organization are most likely to be successful in reducing risk factors related to patient care activities [18].

The other studies among teachers and dentists, interventions aimed at increasing the level of knowledge and awareness, were shown to have a very limited effect on prevention and control of work-related MSD [31,32].

However, there are some positive effects shown by other researchers. Szeto et al. have found positive results in terms of decreased MSC and improved functional outcome measures in the post-intervention period for community nurses, and this positive impact was maintained at the one-year follow-up. The fact that Szeto researched employees with complaints has a significant effect on the outcomes. In our study, nurses, both with and without complaints, were evaluated together [33].

Tullar et al., given the moderate level of evidence, recommended exercise interventions and multi-component patient handling interventions were recommended as practices to consider. Engineering control interventions need to be developed for effective prevention in ICU. They suggest that the intervention programs should include an organizational policy aimed at combating injuries and diseases associated with patient handling and other risky movements [29]. In summary, it may be more effective to consider an ergonomic improvement in which multiple initiatives are carried out together.

Hegewald et al. reviewed ergonomic intervention studies and indicated that the riskiest movement for occupational musculoskeletal injuries was during patient transfer. They defined the neck, waist, and shoulders as the three most affected areas. In our study, the most affected regions were similar. According to the results of the review, they stated that technical aids seem to have a positive effect on preventing musculoskeletal complaints. Still, the level of evidence is not very high [20].

What were the reasons that our program did not give positive results, despite implementing a combined ergonomics intervention program?

First of all, we have to say that, despite a well-designed ergonomics intervention program, we faced a serious restraint in practice. Having only one lifting system which needed to be carried and installed over and over again for each patient was time-consuming and therefore reduced compliance with the program. In the national regulations on ICU, it is proposed that nurses working in tertiary intensive care units should be given responsibility for maximum two patients per nurse [34]. However, it has been found that the requirements of this regulation have not been met in practice (in our case, there were four patients/per nurse). This results in a serious increase in workload and time pressure for nurses. The use of devices may be insufficient due to time pressure and workload. In addition, lack of belief in the effectiveness of the devices and some cultural factors (some impressions from the visit: ‘nothing happens to me’, a fatalistic approach, reluctance to engage in stretching exercises, etc.) influenced adaptation of the initiatives. Kucera et al. investigated this question in nursing professionals. Following this study, non-use of equipment was explained by patient/family preference and reluctance to assist in using the equipment. They also stated further reasons for unwillingness: time restriction, feeling discomfort with the equipment, and the perception that equipment is not necessary [35]. These results show that administrative measures have very important effects on practice. For example, zero-lift campaigns are carried out in different countries to ensure that workers stop lifting patients. A zero-lift concept aims that there will be organizational resources designated for work redesign that incorporate the latest equipment, allowing staff to protect themselves when manual handling during patient care activities [36,37]. Sang et al. also suggested the no lift campaign along with other ergonomics interventions [38]. However, it is important to consider factors such as the weight of the patient and the nurse, and the general condition of the patient (non-cooperative or unconscious). For this purpose, attention should be paid to determining specific norms regarding the use of assistive devices and the number of staff. Another good practice stated by Zaffina et al. is named “disability management” [39]. It has three fundamental components: early detection, adapted accommodations, and high-level internal coordination and communication. Camisa et al. presented a successful “Disability management” implementation with the support of the Ministry of Health at a pediatric healthcare facility [40]. We could have been more successful if we had encouraged stretching exercises along with administrative measures.

Working with a monitor, lifting or transferring dependent patients, kneeling, heavy lifting, and long-standing were the most highlighted ergonomics risk factors precipitating MSD among the nurses in this study. These findings are consistent with the literature indicating manual patient handling or transferring as important factors in MSD among nurses [41,42]. In the clinical results of the study, no significant differences were found between the two groups for any of the MSC variables; even total Cornell scores increased in both groups. Similar to many studies, nurses suffer a relatively high prevalence of complaint: low back, knee, and neck were the three most common body regions during the preceding six months [9,43,44]. Likely, self-reported frequency depends increasingly on awareness of MSD after the intervention.

However, we were not able to prove that multifaceted intervention reduced MSC in nurses. Our study has several strong points. The fact that there was no difference in the variables between the intervention and control groups at the beginning provided an important advantage in evaluating the effectiveness of the interventions. Participation of both intervention and control group nurses was very high. Both groups were successfully followed for 18 months. However, the lack of any detected effect of the intervention might be due to some shortcomings of our study. Furthermore, our intervention was multifaceted, but it still might not have been complex enough to achieve the desired results.

Although the study has provided a detailed understanding of the prevalence of MSCs in ICUs, several limitations still need to be discussed. Firstly, failure to detect any positive effect of the program might be due to the lack of randomization of nurses. It is known that randomization is difficult among the people working in the same department, and there may be interaction and information sharing among them. In our study, randomization could not be performed among the employees, and it was deemed appropriate to design the departments as a control and an intervention group. Therefore, there is a possibility that CG also benefited from the study, which is known as the Hawthorn effect [45]. However, dependent group analysis also failed to reduce significantly MSC and other outcome measures. Therefore, we thought that the bias due to non-random selection had limited or no effects on our results. Furthermore, factors outside of work have not been taken into account, such as lifestyle, social status, and family situation, which have the potential to affect nurses’ health in general. Therefore, our results can only be interpreted with the context limited to the ergonomic interventions, rather than the psychosocial aspects of the MSD. 

The other important issue is related to the small sample size. We did not perform power analysis in the planning. However, post-hoc calculation of the power of the study was moderate. Sample size calculation, assuming an effect size of 0.90 and power of 0.80, based on a 5% difference in musculoskeletal discomfort scores, indicates 21 subjects per group (total = 42) (16). Therefore, we thought that negative or no-effect findings are not mainly due to the small sample size.

Another methodological limitation related to REBA was using on-site direct observation to collect data. It has been recommended that video recording or photographs were superior to direct observation in terms of evaluating risk. This limitation, on the other hand, might have minimal effects on our results since the initial and final assessments have been made by using direct observation.

Moreover, we had some deficiencies in the implementation of interventions in the long term, especially after a year after the initiation of the study. Our study design was mainly focused on workers’ participation. However, we thought that one of the important shortcomings of our study was the limited or non-participation of management. Therefore, we have concluded that interventions, including high-level management participation, would be more beneficial for the development of an ergonomic risk reduction program. This would also boost better cooperation with workers in order to participate in the stretching exercises and the use of auxiliary devices.

## 5. Conclusions

Although the results of our research cannot be generalized to other hospital settings, it is important to show the importance of administrative measures. Our results suggested that interventions without administrative measures may have limited success. Hospital administrations should implement combating strategies, e.g., increase the number of auxiliary devices (where suitable), increase the number of nurses, and ensure or encourage the use of regular breaks in ICUs. Raising self-awareness and encouraging appropriate physical activity, including stretching exercises, and teaching nurses with MSD how to protect themselves appear to be auxiliary or supportive to managerial control and prevention in ergonomic risk factors. The ergonomic training practices should be applied at all levels of the education of nurses. In studies to be carried out, all factors that affect the compliance of the employees with the interventions should be evaluated together. There is a need for randomized controlled trials with a larger number of cases where managerial measures are planned and implemented.

## Figures and Tables

**Table 1:** Basic characteristics of the study population.

Variables	IG N: 35 (%)	CG N: 29 (%)	*p*-Value
Age			
mean ± SD	31.06 ± 5.5	33.6 ± 5.7	0.070
(min–max)	(23–45)	(26–49)	
Gender			
Male	7 (20.0)	3 (10.3)	0.327 *
Female	29 (80.0))	25 (89.7)	
Smoking status			
Current smoker	12 (34.3)	18 (62.19	0.064 (χ^2^ 5.48; df 2)
Ex-smoker	2 (5.7)	2 (6.9)	
Never smoked	21 (60.0)	9 (31.0)	
Chronic diseases			
Yes	6 (17.1)	4 (13.8)	0.722 (X^2^ 0.12; df 1) Phi 0.80
No	29 (82.9)	25 (86.2)	
Previous Ergonomics training			
Yes	19 (54.3)	9 (31.0)	0.166 (χ^2^ 1.92; df 1) (Phi 0.20)
No	16 (47.5)	18 (62.1)	
Working time in ICU (years)			
mean ± SD (min–max)	6.23 ± 5.7 (<1–24)	8.9 ± 5.9 (<1–28)	0.136
Regular rest break use			
Yes	6 (17.1)	4 (13.8)	0.722 (X^2^ 0.12; df 1) Phi 0.80
No	29 (82.9)	25 (86.2)	
Regular exercise			
Yes	15 (42.9)	6 (20.7)	0.585 *
No	20 (57.1)	23 (79.3)	

* Fisher test.

**Table 2:** Findings of musculoskeletal well-being parameters and C MSDQ scores in the last six months.

Variables	IG N: 35 (%)	CGN: 29 (%)	*p*-Value
Visiting a doctor with any MSC			
Yes No	15 (42.9) 20 (57.1)	14 (48.3) 15 (51.7)	0.856 (X^2^ 0.03 df 1) (Phi -0.54)
Use medicine due to any MSD			
Yes No	22 (62.9) 13 (37.1)	19 (65.5) 10 (34.5)	0.218 (X^2^ 1.51 df 1) (Phi -0.18)
Sick leave absence day			
Yes No	6 (17.1) 29 (82.9)	6 (20.7) 23 (79.3)	0.968 (X^2^ 0.002 df 1) (Phi -0.45)
Number of sick leave absence day			
mean ± SD (min–max)	9.8 ± 11.7 (2–30)	16.6 ± 12.5 (3–30)	0.237
Cornell score			0.989
mean ± SD (min–max)	141.9 ± 147.9 (0–638)	176.1 ± 203.6 (0–808)	
Cornell neck			0.711
mean ± SD (min–max)	69.0 ± 81.0 (0–244.0)	10.3 ± 40.1 (0–60.0)	
Cornell lower			0.789
mean ± SD (min–max)	69.0 ± 81.0 (0–244.0)	73.8 ± 98.6 (0–410.0)	
Cornell back			0.978
mean ± SD (min–max)	30.6 ± 42.5 (0–180.0)	10.3 ± 15.7 (0–60.0)	
Cornell upper			0.533
mean ± SD (min–max)	28.4 ± 44.9 (0–180.0)	41.8 ± 59.3 (0–240.0)	

**Table 3:** Rapid Entire Body Assessment (REBA) risk assessment and self-risk assessment by nurses.

Variables	IG N: 35 (%)	CG N: 29 (%)	*p*-Value
**REBA risk assessment**			
Working with computer			
Mean ± SD (min–max)	4.81 ± 4 2–10	6.11 ± 6 (3–8)	0.972
Turning the patients			
Mean ± SD (min–max)	8.7 ± 2.0 5–12	9.7 ± 1.6 (5–12)	0.734
**Self- risk assessment**			
Heavy lifting			
No risk Moderate risk High risk	1 (2.9) 5 (14.3) 29 (82.9)	2 (6.9) 5 (17.2) 22 (75.9)	0.330 (X^2^ 3.42 df 3)
Long-standing			
No risk Moderate risk High risk	1 (2.9) 6 (17.1) 28 (80.0)	2 (6.9) 4 (13.7) 23 (79.3)	0.290 (X^2^ 4.97 df 4)
Bending down			
No risk Moderate risk High risk	1 (2.9) 5 (14.3) 29 (82.9)	2 (6.9) 2 (6.9) 21 (72.4)	0.208 (X^2^ 5.88 df 4)
Pulling—pushing			
No risk Moderate risk High risk	1 (2.9) 7 (20.0) 27 (77.1)	3 (10.3) 5 (17.2) 21 (72.4)	0.256 (X^2^ 5.32 df 4)

**Table 4:** Within-group analysis of nurses’ scores (before and after the intervention).

Variables	IG N: 27 (%)		*p*-Value	CG N: 23 (%)		*p*-Value
	Initial	18th		Initial	18th	
Visiting a doctor with any MSC						
Yes No	16 (59.3) 11 (40.7)	13 (48.1) 14 (51.9)	0.754	13 (56.5) 10 (43.5)	6 (26.0) 17 (73.9)	0.065
Use medicine due to any MSD						
Yes No	16 (59.3) 11 (40.7)	13 (48.1) 14 (51.9)	0.549	15 (65.2) 8 (34.3)	14 (60.9) 9 (39.1)	1.0
Sick leave absence day						
Yes No	6 (22.2) 21 (77.8)	5 (18.5) 22 (81.5)	1.0	4 (17.4) 19 (82.6)	2 (8.7) 21 (91.3)	0.625
Number of sick leave absence day						
mean ± SD (min–max)	8.83 ± 10.7 (2–30)	8.0 ± 12.3 (2–30)	1.0	18.0 ± 14.2 (2–30)	2.0 ± 0 (2)	0.317

**Table 5:** Between-group analysis in the 18th month (IG vs. CG).

	IG N: 27 (%)	CG N: 23 (%)	*p*-Value
Visiting a doctor with any MSC			
Yes No	13 (48.1) 14(51.9)	6 (26.1) 17 (73.9)	0.190 (X^2^ 1.71 df 1)
Use medicine due to any MSD			
Yes No	13 (48.1) 14 (51.9)	14 (60.9) 9 (39.1)	0.539 (X^2^ 0.37 df 1)
Sick leave absence day			
Yes No	5 (18.5) 22 (81.5)	2 (8.7) 21 (91.3)	0.430 *
Number of sick leave absence day			
mean ± SD (min–max)	8.0 ± 12.3 (2–30)	2.0 ± 0 (2)	0.324
Self- risk assessment			
Heavy lifting			
No risk Moderate risk High risk	- 1(3.7) 26(96.3)	- 3(13.0) 20(87.0)	0.322 *
Long-standing			
No risk Moderate risk High risk	- 1(3.7) 26(96.3)	- 3(13.0) 20(87.0)	0.588 *
Bending down			
No risk Moderate risk High risk	- 1(3.7) 26(96.3)	- 3(13.0) 20(87.0)	0.322 *
Pulling—pushing			
No risk Moderate risk High risk	- 1(3.7) 26(96.3)	1(4.3) 3(13.0) 19(82.6)	0.248 (X^2^ 2.78 df 2)

* Fisher Test.

**Table 6:** Two-factor repeated measures ANOVA results.

Cornell Score	IG N: 27	95% CI	CG N: 23	95% CI	Between-Group Analysis *p*-Value
Initial (mean) (min–max)	207.5 (0–790)	119.6–295.5	222.1 (0–790)	126.8–317.3	Between groups: 0.641 Interaction: 0.992
18 th (mean) (min–max)	292.3 (27–780)	213.0–371.6	281.2 (84–752)	195.3–367.1	
Within group analysis *p*-Value	<0.001		<0.001		
